# Development of an IgY-based lateral flow immunoassay for detection of fumonisin B in maize

**DOI:** 10.12688/f1000research.19643.2

**Published:** 2019-12-12

**Authors:** Tien Viet Tran, Binh Nhu Do, Thao Phuong Thi Nguyen, Tung Thanh Tran, Son Cao Tran, Ba Van Nguyen, Chuyen Van Nguyen, Hoa Quang Le

**Affiliations:** 1 Vietnam Military Medical University, Hanoi, 100000, Vietnam; 2 School of Biotechnology and Food Technology, Hanoi University of Science and Technology, Hanoi, 100000, Vietnam; 3 Laboratory of Food Toxicology and Allergens Testing, National Institute for Food Control, Hanoi, Vietnam

**Keywords:** fumonisin B, rapid methods, lateral flow immunoassay, IgY

## Abstract

Fumonisins are among the most prevalent mycotoxins in maize, causing substantial economic losses and potential health risks in humans and animals. In the present study, in-house polyclonal IgY antibody against fumonisin B1 (FB _1_) and B2 (FB _2_) was applied for the development of a competitive lateral flow immunoassay detecting these mycotoxins in maize grains with the limit of detection of 4000 µg/kg, which corresponds to the maximum residue limit adopted by the European Commission. To this end, factors affecting the test performance including nitrocellulose membrane type, dilution factor of maize homogenates in running buffer, amount of detection conjugate, and incubation time between detection conjugate and samples were optimized. Under the optimal condition (UniSart ^®^ *CN140 *nitrocellulose membrane, FB _1_-BSA immobilized at 1 µg/cm, 1:10 dilution factor, 436 ng of gold nanoparticle conjugate, 30 minutes of incubation), the developed test could detect both FB _1_ and FB _2_ in maize with limit of detection of 4000 µg/kg, and showed no cross-reactivity to deoxynivalenol, ochratoxin A, aflatoxin B1 and zearalenone. When applied to detect FB _1_ and FB _2 _in naturally contaminated maize samples, results obtained from the developed assay were in good agreement with those from the high-performance liquid chromatography method. This lateral flow immunoassay is particularly suitable for screening of fumonisins in maize because of its simplicity and cost-effectiveness.

## Introduction

Fumonisins are a group of mycotoxins from *Fusarium* species, mostly *Fusarium proliferatum* and *Fusarium verticillioides* ( Scott, 2012). To date, four groups of fumonisin have been identified (A, B, C and P-series), among which fumonisin B _1_ (FB _1_) and fumonisin B _2_ (FB _2_) are the most common mycotoxins found in corn, and were found to have various toxic and carcinogenic effects ( Munkvold *et al.*, 2019; Scott, 2012). For instance, highly significant associations between intake of fumonisin-contaminated maize and oral cancer, pharyngeal cancer, and esophageal cancer have been observed ( Alizadeh *et al.*, 2012; Franceschi *et al.*, 1990; Sun *et al.*, 2007). Equine leukoencephalomalacia and porcine pulmonary edema have also been revealed to be related to consumption of fumonisin-contaminated maize ( Haliburton & Buck, 1986; Marasas, 2001). Due to its toxicity, the European Commission has adopted the maximum residue limit (MRL) for the presence of total fumonisins (as the sum of FB _1_ and FB _2_) in raw maize at 4000 µg/kg ( EC, 2007).

Recent studies pointed out fumonisin contamination in corn represents a major public-health concern in diverse countries including China ( Fu *et al.*, 2015; Guo *et al.*, 2016; Hu *et al.*, 2019; Liu *et al.*, 2017), Brazil ( Scussel *et al.*, 2014), Kenya ( Mutiga *et al.*, 2015), South Africa ( Mngqawa *et al.*, 2016), Malawi ( Mwalwayo & Thole, 2016), Tanzania ( Kamala *et al.*, 2016), Nigeria ( Chilaka *et al.*, 2016), Ethiopia ( Getachew *et al.*, 2018), Somalia ( Wielogorska *et al.*, 2019). In Vietnam, Hieu Phuong *et al*. (2015) showed that FB _1_ and FB _2_ were the major mycotoxin that contaminated maize with 67% of incidence, a range of positive samples for FB _1_ and FB _2_ at 102 to 10799 µg/kg and 102–5051 µg/kg respectively.

Conventionally, chromatography methods such as high performance liquid chromatography (HPLC) or liquid chromatography tandem mass spectrometry (LC-MS/MS) could be used to detect fumonisins in maize ( Cigić & Prosen, 2009; Gruber-Dorninger *et al.*, 2018). However, they are laborious, time consuming and require specialized equipment. On the other hand, lateral flow immunoassays (LFIAs) are cost-effective, easy to use and suitable for on-site analysis. Several LFIAs have been developed for quick and simple screening of fumonisins in various types of sample ( Anfossi *et al.*, 2010; Venkataramana *et al.*, 2014; Wang *et al.*, 2013; Wang *et al.*, 2014; Yu *et al.*, 2015). Nonetheless, most available LFIAs today are based on monoclonal or polyclonal IgG from mammals, which increases the cost of production and involves ethical issues of animal welfare.

Polyclonal IgY antibodies from egg yolk of laying hens represent an attractive alternative to monoclonal and rodent polyclonal antibodies. With one course of immunization, IgY could be extracted non-invasively in a large quantity (up to 40–80 mg), with 2–10% of which being antigen specific ( Kovacs-Nolan & Mine, 2004; Pauly *et al.*, 2011). As a result, IgY has been increasingly employed for the development of rapid tests. Its usefulness in LFIAs has been demonstrated for detection of morphine ( Gandhi *et al.*, 2009), methicillin-resistant *Staphylococcus aureus* ( Yamada *et al.*, 2013), staphylococcal enterotoxins ( Jin *et al.*, 2013), and rhein ( Zhang *et al.*, 2018b ).

In the present study, we demonstrated the development of a IgY-based LFIA for simple and cost-effective screening of total fumonisins (as the sum of FB _1_ and FB _2_) in raw maize with LOD equal to the MRL of 4000 µg/kg.

## Methods

### Preparation of FB _1_-BSA conjugate

 Conjugation of FB _1_ to BSA was performed following the protocol by Szurdoki *et al*. (1996) with some modifications. Glutaraldehyde (GA) solution 50 % (W/V) (Sigma-Aldrich, Cat Nº 340855) was used as the cross-linker reagent while BSA (Sigma-Aldrich, Cat Nº A9085) was used as the carrier protein. Specifically, BSA (5 mg/mL) was dialyzed in 20 mM sodium phosphate buffer pH 6.0. A total of 10 µl of GA 50% (W/V) were then incubated with 1 mL of the dialyzed BSA solution overnight at room temperature. After incubation, excess GA was removed by dialyzing in Phosphate-buffered saline (PBS), followed by addition of 1 mg of FB _1_ (Santa Cruz Biotechnology, Cat Nº sc-201395A) to achieve the molar ratio of 20:1 (FB _1_:BSA-GA). The mixture was incubated at 4°C overnight on a Dynal Biotech rotary shaker (10 rpm) before the addition of 80 µl of glycine 1 M (Bio Basic, Cat Nº GB0235) to block unreacted aldehyde groups. The reaction mixture was further incubated at room temperature for 4 hours. Subsequently, sodium borohydride powder (Sigma-Aldrich, Cat Nº 452882) was added to the mixture (final concentration of 10 mg/mL) and incubated for 4 hours at room temperature. The obtained solution was then dialyzed and concentrated in 10 mM Borat buffer pH 8.5 using a 10 kDa Amicon ^®^ Ultra-4 Centrifugal Filter Unit (Millipore, Cat Nº UFC801024). Lastly, FB _1_-BSA conjugate was quantified using Nano Drop 2000 (Thermo Fisher Scientific) and stored at 4 °C.

### Production of IgY against FB _1_-KLH

***Animal procedures.*** All animal procedures were approved by the Research Ethics Committee of Vietnam Military Medical University (Decision No. 29/2015/HVQY-HD3). All efforts were made to ameliorate harm to the animals, by conforming to the Principles of animal care and use in research adopted by the Vietnam Military Medical University.

A total of two Fayoumi hens (aged 20 weeks) were sourced from Thuy Phuong Poultry Research Center, Vietnam for IgY production. Hens were housed individually in standard battery cages (800 cm ^2^/hen) and received commercial rations (A55, Anova Feed) and water *ad libitum*. The temperature was kept between 25 and 35°C and the light cycle was 16 hours light/8 hours dark.

Polyclonal IgY antibody against FB1-KLH was obtained as described previously ( Do *et al.*, 2016). Briefly, FB1-KLH was prepared according to the procedure described by Szurdoki *et al*. (1996). Glutaraldehyde (GA) solution 50 % (W/V) (Sigma-Aldrich, Cat Nº 340855) was used as the cross-linker reagent. A total of 10 mg of KLH (Thermo Fisher Scientific, Cat Nº 77600) was dissolved in 12 mL of water and dialyzed against 2 L of 0.2% GA in 0.01 M PBS (pH 7.5) for 20 hours. Excess GA was removed by dialyzing in PBS, followed by dropwise addition of 2 mg of FB1 (Santa Cruz Biotechnology, Cat Nº sc-201395A). The mixture was incubated at 4°C overnight on a Dynal Biotech rotary shaker (10 rpm) before the addition of 80 µl of glycine 1 M (Bio Basic, Cat Nº GB0235) to block unreacted aldehyde groups. The reaction mixture was further incubated at room temperature for 4 hours. The obtained solution was then dialyzed and concentrated in PBS pH 7.5 using a 100 kDa Amicon ^®^ Ultra-4 Centrifugal Filter Unit (Millipore, Cat Nº UFC810024). Lastly, FB1-KLH conjugate was quantified using Nano Drop 2000 (Thermo Fisher Scientific) and stored at 4 °C.

The chickens were intramuscularly immunized three times in 10 days intervals to elicit strong immune response. For the first immunization, an injection dose of 1.0 mL was prepared by mixing 0.2 mg of FB _1_-KLH with an equal volume of complete Freund's adjuvant (Sigma-Aldrich, Cat Nº F5881). For the two subsequent booster immunizations, the amount of immunogen was decreased to 0.1 mg of FB _1_-KLH and incomplete Freund's adjuvant (Sigma-Aldrich, Cat Nº F5506) was used. Eggs were collected two weeks after the last immunization and stored at 4°C. The extraction of IgY was performed by polyethylene glycol (PEG) (Sigma-Aldrich, Cat Nº 81255) precipitation as described by Pauly *et al*. (2011). The eggshell was carefully cracked, and the yolk was transferred to a “yolk spoon” and filter paper to remove egg white. The egg yolk skin membrane was cut before the yolk was poured into a 50 ml tube. Twice the egg yolk volume of PBS was added to the tube and mixed by vortexing. PEG 6000 was added to achieve the final concentration of 3.5 % (w/v) and the tube was vortexed and rolled for 10 minutes on a Dynal Biotech rotary shaker (30 rpm) before being centrifuged at 8000 *× g,* 4 °C for 10 minutes. The supernatant was subjected to filtration and then to precipitation of IgY by adding PEG 6000 (final concentration 12 % (w/v)). The tube was vortexed and centrifuged at 8000 *× g,* 4 °C for 30 minutes and the supernatant was discarded. The pellet was dissolved in 10 mL PBS and PEG 6000 was added to achieve the final concentration of 12 % (w/v). Subsequently, the tube was centrifuged at 8000 *× g,* 4 °C for 30 minutes. The pellet was dissolved in 5 mL of PBS and IgY was further purified by microfiltration via a 0.45 μm membrane and ultrafiltration using 100 kDa Amicon® Ultra-4 Centrifugal Filter Units (Millipore, Cat Nº UFC810008). Finally, IgY was stored at -80°C in small aliquots.

### Preparation of IgY-conjugated gold nanoparticles

IgY was conjugated to gold nanoparticles via covalent immobilization, following instructions of BioReady 40 nm Carboxyl Gold (Nanocomposix, Cat Nº AUXR40-5M). The procedure involves linking the primary amine groups of the antibody to the carboxylic groups on the surface of the particles by the use of EDC/Sulfo-NHS coupling chemistry.

Specifically, before conjugation, 10 mg/mL EDC (Sigma-Aldrich, Cat Nº 03449) and 10 mg/mL Sulfo-NHS (Sigma Aldrich, Cat Nº 56485) were freshly prepared in H2O; and the polyclonal IgY antibody was dialyzed in 10 mM potassium phosphate (pH 7.4) using Amicon Ultra-0.5 Centrifugal Filter Unit (Millipore, Cat Nº UFC501096). One milliliter (0.83 mg) of BioReady 40 nm Carboxyl Gold (NanoComposix, Cat Nº AUXR40-5M) was mixed with 20 µl and 40 µl of the prepared EDC and Sulfo-NHS respectively. The mixture was then incubated on a Dynal Biotech rotary shaker (15 rpm) at room temperature for 30 minutes then centrifuged at 3600 × g for 10 minutes. The supernatant was then removed completely, and the gold nanoparticles were resuspended in 1 mL of Reaction Buffer (5 mM potassium phosphate, 0.5 % 20K MW PEG, pH 7.4). The reaction tube was then incubated with 50 µg of IgY on a Dynal Biotech rotary shaker (15 rpm) at room temperature for 2 hours. Subsequently, blocking of remaining NHS-esters was performed using 10 µl of 50% (w/v) hydroxylamine. IgY-conjugated gold nanoparticles were then washed three times with 1 mL of Reaction Buffer and resuspended in 10 mL of Conjugate Diluent (0.1X PBS, 0.5% BSA, 0.05% Sodium Azide) and stored at 4°C.

### Preparation of LFIA test strips

Test strips were prepared following Posthuma-Trumpie *et al*. (2008) with some modifications. Briefly, a Linomat V (Camag, Cat Nº 022.7808) was used to dispense FB _1_-BSA and Mouse monoclonal 0.8C Anti-Chicken IgY H&L (Abcam, Cat Nº ab82229) at the test line and control line positions of a nitrocellulose membrane respectively. For the control line, immunoglobulins were dispensed at a dose of 0.5 µg/cm, at the position of 2 cm away from the dipping point. For the test line, FB _1_-BSA was dispensed at a dose of 1 µg/cm at the position of 1.5 cm away from the dipping point. The membrane was then dried for 2 hours at 37°C. A second plastic backing and an absorption pad (Extra Thick Blot Paper, BIO-RAD, Cat Nº 1703969) were applied; and the membranes were cut into 4 mm-wide test strips using an Autokun cutter (Hangzhou Autokun Technology). Test trips were sealed in aluminum packages with a desiccation pad and stored at 4°C until use. Three different membranes were tested, namely *CNPC-SS12, 10 µm* with wicking time of 140 ± 28s/40 mm (MDI technologies, Cat Nº CNPC-SS12-10µm-25mm), UniSart® CN140 with wicking time of 95-155s/40 mm (Sartorius, Cat Nº 1UN14ER100025NTB), and UniSart® CN 95 with wicking time of 65-115s/40 mm (Sartorius, Cat Nº 1UN95ER100040WS).

### Sample preparation and assay procedures

Blank and naturally contaminated maize grains were collected from local markets in Hanoi, Vietnam during the year of 2017. The samples were finely ground using an A 11 basic Analytical mill (IKA) and a 500 µm sieve.

Spiking of FBs into maize was performed on a blank sample. Briefly, 5 g of ground maize were spiked with 10–40 µl of FB _1_ or FB _2_ stock solution of 1 mg/mL to achieve final content of 2000 – 8000 µg/kg. Spiked samples were left 24 hours at 4°C. Extraction of FBs and LFIA analysis were performed as described below.

**Figure 1.**
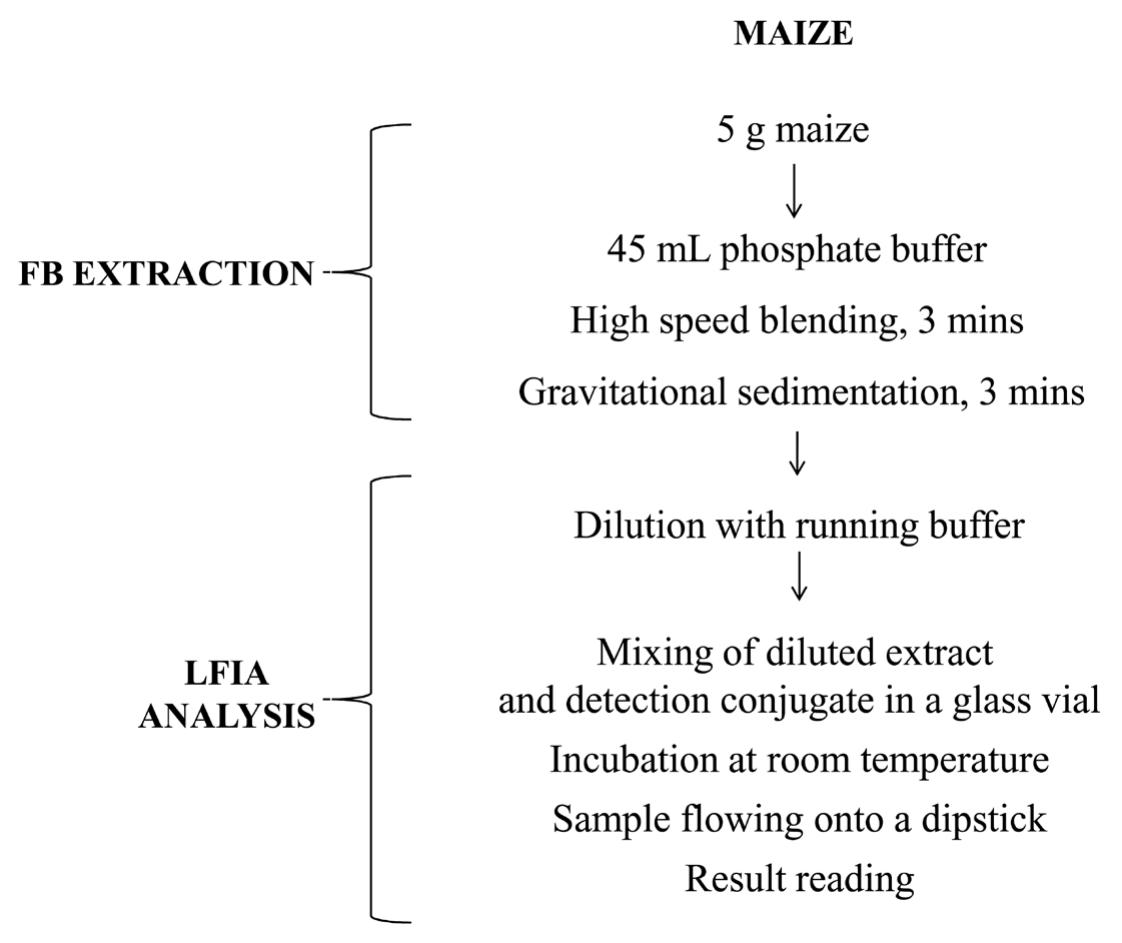
Schematic representation of fumonisin extraction and lateral flow analysis for maize samples. A total of 5 g of maize were homogenized in 45 mL of phosphate buffer for 3 minutes. The mixture was allowed to settle for 3 minutes before collection of the supernatant, which was further diluted in running buffer. A hundred microliters of the diluted extract were used for lateral flow immunoassays (LFIA) analysis. After being incubated with detection conjugate at room temperature, samples were flowed onto LFIA strips. Results were read with the naked eye after the strips absorbed fluid completely.

The protocol for FB extraction from naturally contaminated or spiked samples ( Figure 1) was based on the work of Pietri & Bertuzzi (2011) and Lattanzio *et al*. (2012). Instead of using organic solvents, FB was extracted with 0.4 M phosphate buffer (PB) at pH 7.5 ( Pietri & Bertuzzi, 2011). Specifically, 5 g of maize flour were mixed with 45 mL of PB and blended using a T10 basic ULTRA-TURRAX ^®^ (IKA) at the highest speed for 3 minutes. The blended samples were then allowed to settle for 3 minutes to recover the supernatant, which was further diluted 1:3, 1:5, 1:10 or 1:20 in Running Buffer (100 mM Borat Buffer, 0.5 % BSA, 0.05% Tween ^®^-20, 0.02 % NaN _3_, pH 8.5). For LFIA analysis, 100 µl of the diluted extracts were dispensed into a 2-mL lyophilization glass vials and incubated with 174 ng, 436 ng or 697 ng (corresponding to 2, 5, 8 µl) of detection conjugate for 0 to 60 minutes before being flowed vertically onto LFIA test strips. After 25 minutes, results could be read with the naked eye or captured by a Perfection V600 scanner (Epson). Optical densities of test lines and control lines were digitalized to obtain signal values using ImageJ software (ver.1.47) ( Schneider *et al.*, 2012). GraphPad Prism 6.0 (GraphPad Software Inc.) was used to statistically analyze and graph the data. Unpaired, two-tailed *t-*tests were performed to determine statistical significance.

### Specificity test

To test the specificity of the developed assay, four following mycotoxins at 100-fold and 1000-fold of the maximum residue limit (MRL) (deoxynivalenol at 1750 ng/mL and 17500 ng/mL; ochratoxin A at 5 ng/mL and 50 ng/mL; aflatoxin B _1_ at 10 ng/mL and 100 ng/mL; and zearalenone at 350 ng/mL and 3500 ng/mL) were spiked into diluted extracts of blank samples. All of these mycotoxins were purchased from FERMENTEK Ltd (Cat Nº: 51481-10-8, 303-47-9, 1162-65-8, 17924-92-4). The subsequent LFIA analyses were performed as mentioned above.

### Quantification of FB _1_ and FB _2_ in maize using HPLC-MS method

FBs in maize were quantified by the EN 13585:2001 standard method ( CEN, 2001) with some modifications. Briefly, 5g of maize were mixed with 5 mL of methanol-water and blended for 5 minutes using a T10 basic ULTRA-TURRAX® (IKA). Maize extracts were then collected by centrifugation (500 *× g*, 10 minutes) and filtering (Whatman) and 1 mL aliquot of filtrates was loaded into a preconditioned Bond-Elut strong-anion-exchanging cartridge (Agilent, Cat Nº 14102017). After washing with methanol, elution was performed using 10 mL of methanol-acetic acid (99:1 v/v). The eluate was then evaporated under a stream of nitrogen, washed with 1 mL of methanol and evaporated again. Dried samples were reconstituted in 1 mL of methanol before HPLC-MS/MS analysis. HPLC injection (10 µl) was performed on a system consisting of a Shimadzu LC-20ADVP pump; a Symmetry HPLC column (150 mm × 3.0 mm i.d. × 3.5 µm) maintained at 30°C (Waters, 186000695); and a SCIEX Triple Quad™ 5500 mass spectrometer. The analytical separation was performed with water-acid formic (99.9-0.1, v/v) and acetonitrile as mobile phases A and B respectively. The gradient elution program began with an isocratic step of 80:20 A:B for 2 minutes and then increased linearly to 10:90 A:B over 5 minutes, which was maintained for 3 minutes, and returned to the starting condition. The condition was then held constant for 3 minutes. The flow rate was kept at 0.5 mL/min. The HPLC column effluent was pumped to the MS/MS system, with the electrospray ionization (ESI) probe operating in positive mode. The following parameters were used: capillary voltage, 5000 V; desolvation gas temperature, 450°C; ion source gas 1 and gas 2 pressure, 40 and 30 psi, respectively. Detection was carried out in multiple reaction monitoring (MRM) mode with two transitions for each compound. Nitrogen was used as the collision gas, and the collision cell pressure was 7 psi. The reference standards of FBs were purchased from LGC Standards (Cat Nº B-MYC0400-C and B-MYC0420-1).

## Results

### Development and optimization of LFIA

The LFIA developed in the present study is based on the competitive format in which polyclonal IgY antibody, showing recognition specificity toward both FB1 and FB2 ( Do *et al.*, 2016), is conjugated to gold nanoparticle (see underlying data ( Tran *et al.*, 2019)). The labeled antibody was mixed with the sample extract in a glass vial, and the mixture was incubated to allow antigen-antibody complexes to form before flowing onto the nitrocellulose membrane which contains a test line and a control line. In our assay, FB _1_-BSA conjugate was immobilized on the test line while a secondary antibody against chicken IgY was coated on the control line. In a negative sample, the free detection antibody binds to the FB _1_-BSA conjugate immobilized on the test line, forming a visible line. An excess of the labeled antibody migrates to the control line and binds to the secondary antibody. As a result, a negative sample will form two visible lines on the nitrocellulose membrane. In a positive sample, FBs in the sample extract will react with all of the available binding sites of the antibody, thus preventing attachment of the detection antibody to the FB _1_-BSA conjugate on the test line. All of the detection conjugate will migrate to the control line and will form a visible line. Consequently, a positive sample will form only one line at the control zone.

Optimization has been performed with FB _1_, the most common mycotoxin in maize, so that the samples with FB _1_ concentration equal to or beyond the maximum residue limit of 4000 µg/kg, will result in no visible line at the test zone. To this end, the effects of nitrocellulose membrane type, dilution factor of maize homogenates in running buffer, amount of detection conjugate, and the incubation time between sample extract and detection conjugate, on the test performance were evaluated.

**Figure 2.**
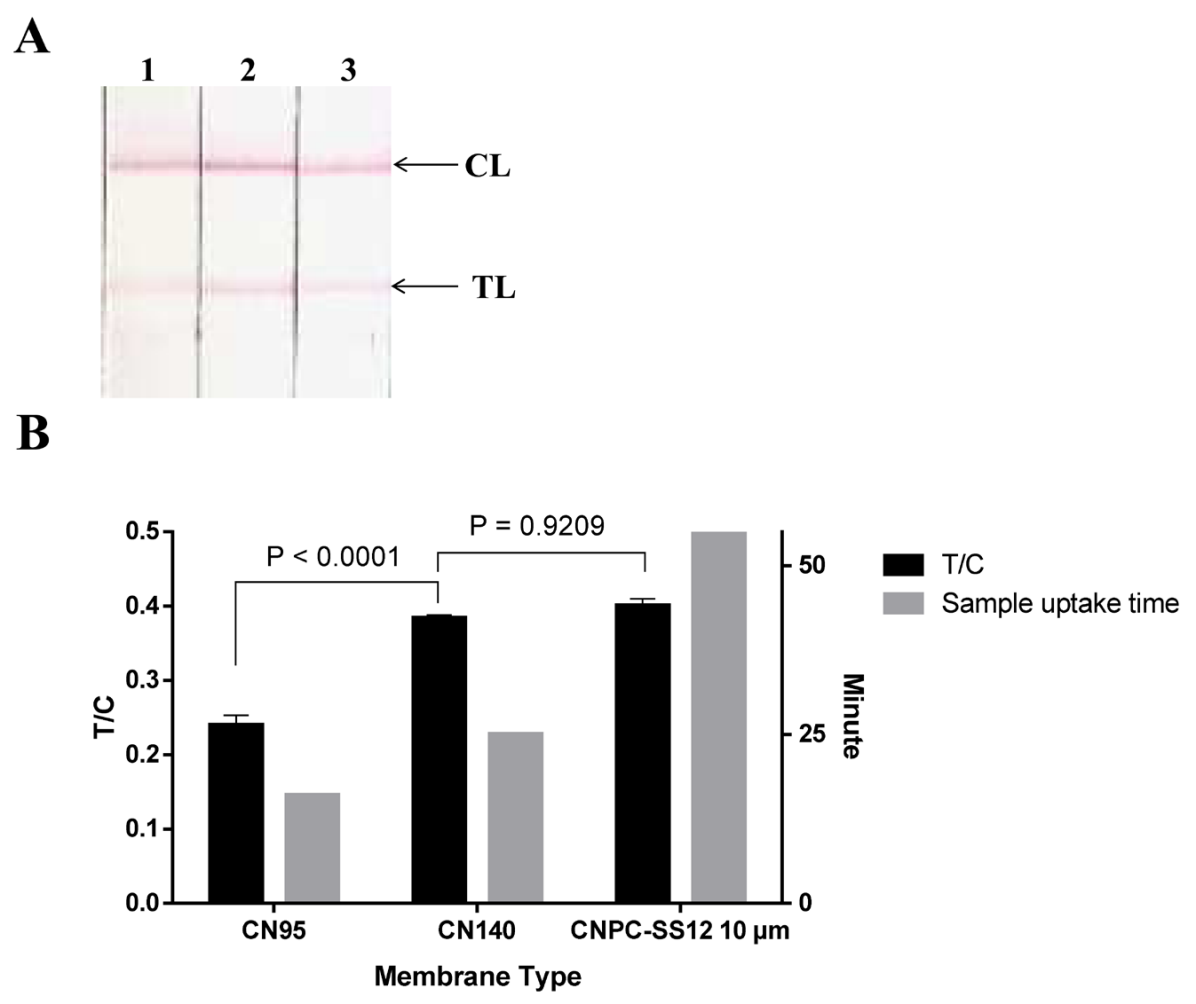
Selection of nitrocellulose membrane. ( **A**) Images of negative controls (0 µg/mL fumonisin group B (FB)) on three different nitrocellulose membranes; 1, UniSart ^®^ *CN95* (Sartorius) *, 2,* UniSart ^®^ *CN140* (Sartorius); 3, *CNPC-SS12 10 µm* (MDI technologies); CL, control line; TL, test line. ( **B**) Quantification of signal intensities and sample uptake time for each type of membrane. Sample uptake time is defined as the total time required for membranes to absorb fluid completely. T, test line signal; C, control line signal.

***Selection of nitrocellulose membrane.*** Flow rate and protein-binding capacity of nitrocellulose membranes directly affect sensitivity and run time of a LFIA ( O’Farrell, 2008). Generally, nitrocellulose membranes with a low flow rate will facilitate the formation of immunocomplexes at the test and control lines. However, it could lead to extended run times and false positive results ( O’Farrell, 2008). In the present study, selection of nitrocellulose membrane was carried out by analyzing running buffer mixed with detection conjugate (negative controls) on three different nitrocellulose membranes. Figure 2 indicated that UniSart ^®^ *CN140* (Sartorius) and *CNPC-SS12, 10 μm* (MDI technologies) produced higher signal intensities than UniSart ^®^ *CN95* (Sartorius). Although the difference in signal intensity between UniSart® CN140 and *CNPC-SS12, 10 μm* was not statistically significant (p = 0.9209), sample uptake time was significantly lower on UniSart® CN140 ** ( Figure 2B). Therefore, UniSart ^®^ *CN140* from Sartorius was chosen for subsequent experiments.

**Figure 3.**
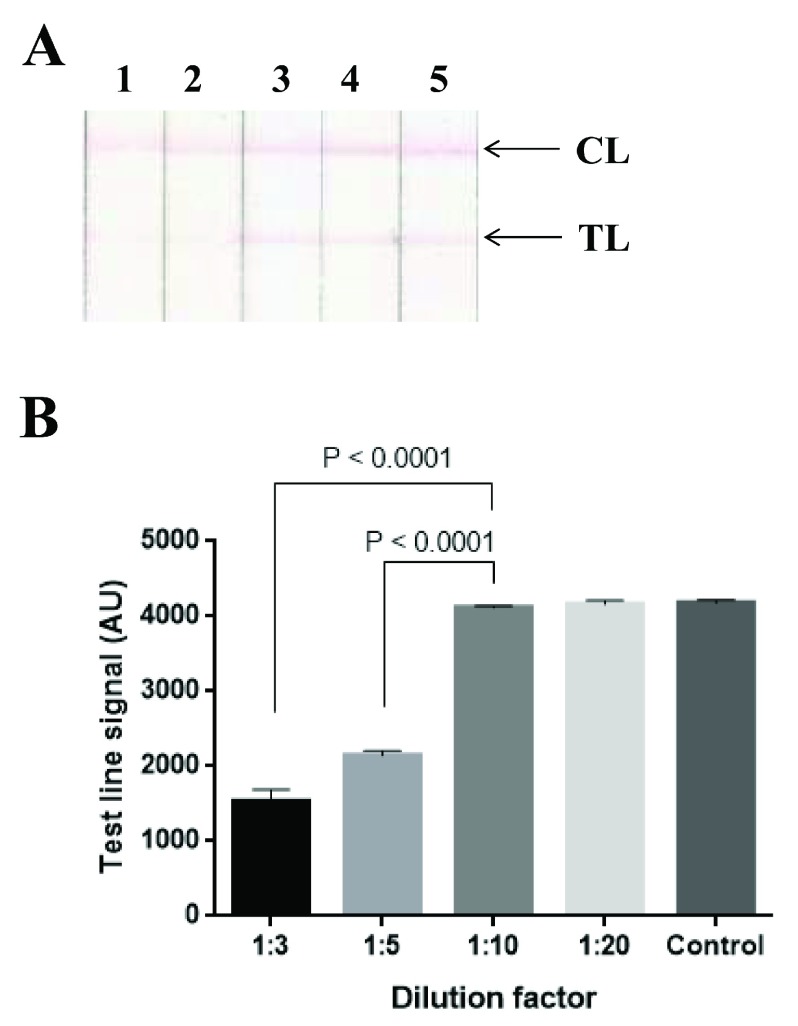
Optimization of dilution factor of maize extract. ( **A**) Images of diluted extracts of blank maize samples (negative with fumonisin group B (FB) by high performance liquid chromatography (HPLC)) with different dilution factors on representative lateral flow immunoassay strips; 1, 2, 3, 4, maize homogenates diluted 3-, 5-, 10-, 20-fold respectively in running buffer; 5, negative controls. CL, control line; TL, test line. ( **B**) Quantification of test line signals. Control, negative control; AU, arbitrary unit.

***Optimization of dilution factor of maize extract.*** Food sample extracts are commonly diluted before analysis by LFIA to minimize the negative effects of sample matrix on antibody-antigen reactions ( Anfossi *et al.*, 2011; Lattanzio *et al.*, 2012). To determine the optimal dilution factor, blank maize grains were subjected to extraction using phosphate buffer (PB) and dilution in running buffer with ratios ranging from 1:3 to 1:20. According to Pietri & Bertuzzi (2011), average recovery percentages were 95.5±1.9% and 96.7±2.1% for FB _1_ and FB _2_ when extracted in PB. Furthermore, this extraction method does not require the use of toxic solvents and it may prevent possible inhibiting effects of organic solvents on antibody-antigen reaction ( Rehan & Younus, 2006; Russell *et al.*, 1989). Test line intensities generated by the diluted extract samples were compared with those from negative controls. Figure 3 revealed that signal intensities were decreased at dilution factors of 1:3 and 1:5, comparing to those from negative controls. However, starting from 1:10 dilution, test line signals were similar to negative controls ( Figure 3B). As a result, an optimal dilution factor of 1:10 was set for subsequent experiments.

***Optimization of amount of detection conjugate.*** Quantity of labeled antibody directly affects the limit of detection of a competitive LFIA. In fact, if a low amount of detection conjugate is used, no visible test lines will be formed even low levels of FBs (less than 4000 µg/kg) are present in the samples. Furthermore, using a low amount of detection conjugate will decrease the signal intensities at both test and control lines, causing difficulties in result interpretation. On the other hand, using an excessive amount of detection conjugate will negatively affect the analytical sensitivity of the assay as more toxins are required to saturate all the binding sites of the detection antibody.

**Figure 4.**
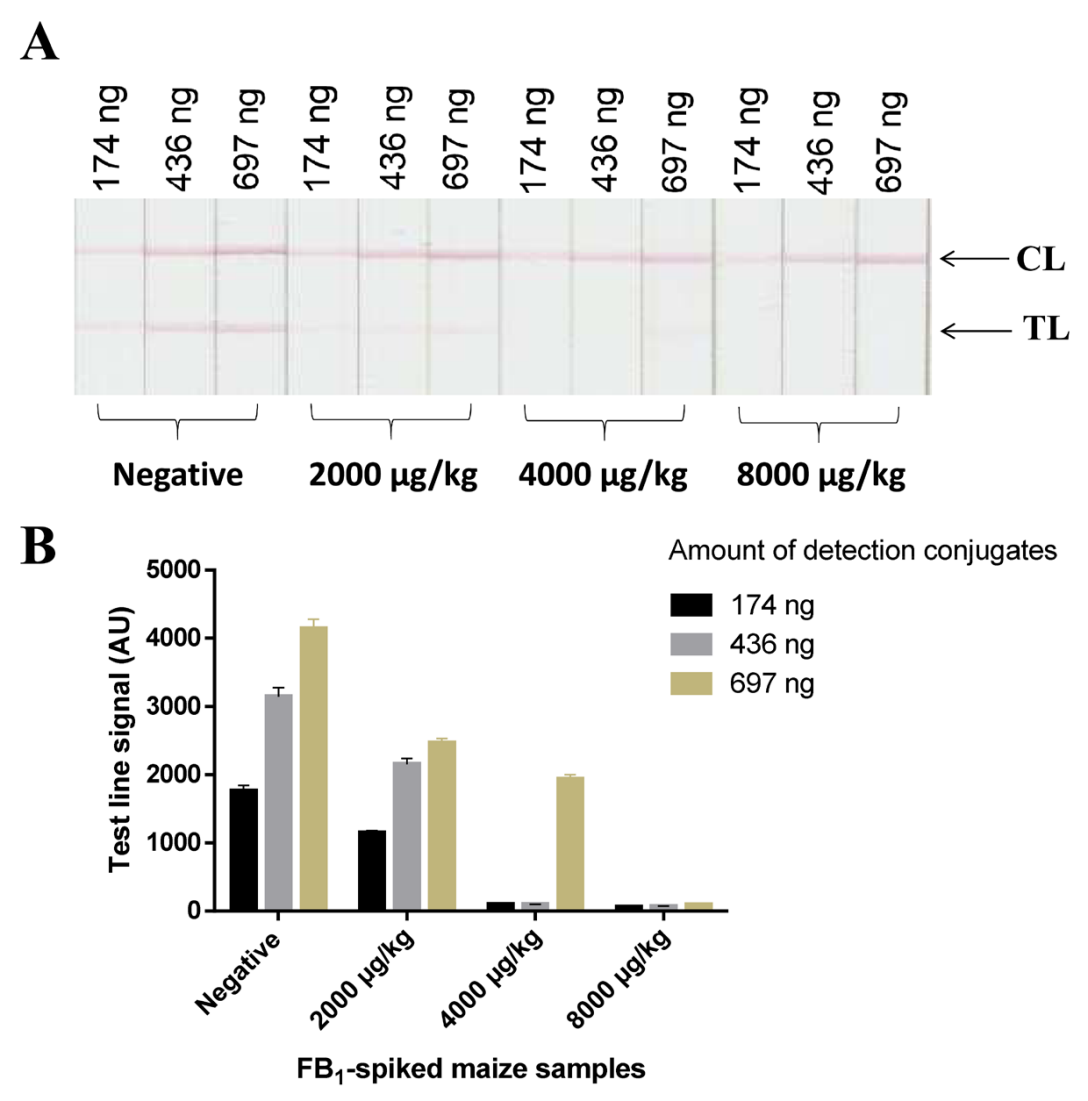
Optimization of amount of detection conjugate to achieve the detection limit of 4000 µg/kg of fumonisin B _1_ (FB _1_). ( **A**) Images of negative controls and diluted extracts of blank maize samples spiked with FB _1_ at 2000 µg/kg, 4000 µg/kg, and 8000 µg/kg using 174 ng, 436 ng or 697 ng of detection conjugate on representative lateral flow immunoassay (LFIA) strips. Maize extracts were incubated with detection conjugate for one hour at room temperature before being analyzed on LFIA strips. Experiment was performed with 8 replicates. CL, control line; TL, test line. ( **B**) Quantification of test line signals. AU, arbitrary unit.

In the present study, various amounts (174 ng, 436 ng, and 697 ng corresponding to 2, 5, and 8 µl) of detection conjugate were used to react with FB _1_ extracted from blank samples spiked with this toxin at 2000 µg/kg, 4000 µg/kg or 8000 µg/kg. For samples spiked with 2000 µg/kg FB _1_, test lines were observed on all test strips regardless of the amounts of detection conjugate used ( Figure 4). Conversely, no test line was observed when FB _1_ is present at 8000 µg/kg ( Figure 4). At the cut-off level of 4000 µg/kg, test line signal was still present when 697 ng of detection conjugate were used while no test line was visible when 174 ng or 436 ng of detection conjugate were used. However, using 174 ng of detection conjugate resulted in low signals of test line on negative controls ( Figure 4). Therefore, 436 ng of IgY-conjugated gold nanoparticles were used for further studies.

**Figure 5.**
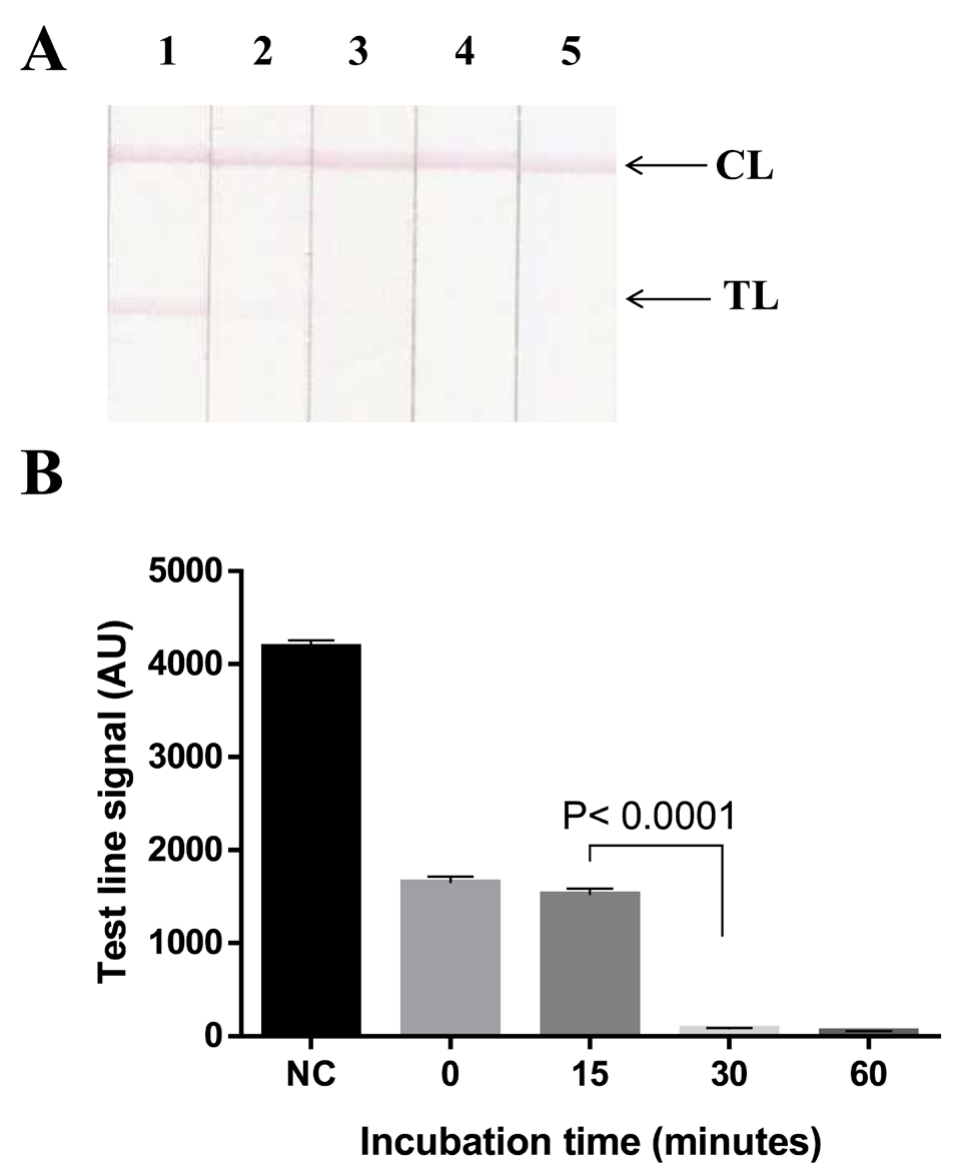
Optimization of incubation time between detection conjugate and samples to be analyzed. ( **A**) Images of diluted extract (from blank sample spiked with fumonisin B _1_ (FB _1_) at 4000 µg/kg) incubated with 436 ng of detection conjugate for 0, 15, 30 and 60 minutes on representative lateral flow immunoassay strips. 1, negative control; 2, 3, 4, 5, incubation time of 0, 15, 30, 60 minutes respectively; CL, control line; TL, test line. ( **B**) Quantification of signal intensities. NC, negative control; AU, arbitrary unit.

***Optimization of incubation time between samples and detection conjugate.*** Effects of incubation step between detection conjugate and FB _1_ at the cut-off level (extracted from blank samples spiked with FB _1_ at 4000 µg/kg) on the test performance were assessed by varying the incubation time from 0 to 60 minutes. Results ( Figure 5) indicated that test lines were still visible when the incubation time was 0 or 15 minutes, while no test lines were observed when the incubation time was 30 or 60 minutes. To shorten the analytical procedure, an incubation time of 30 minutes was chosen.

### Determination of limit of detection of the developed LFIA for FB _2_ in maize

**Figure 6.**
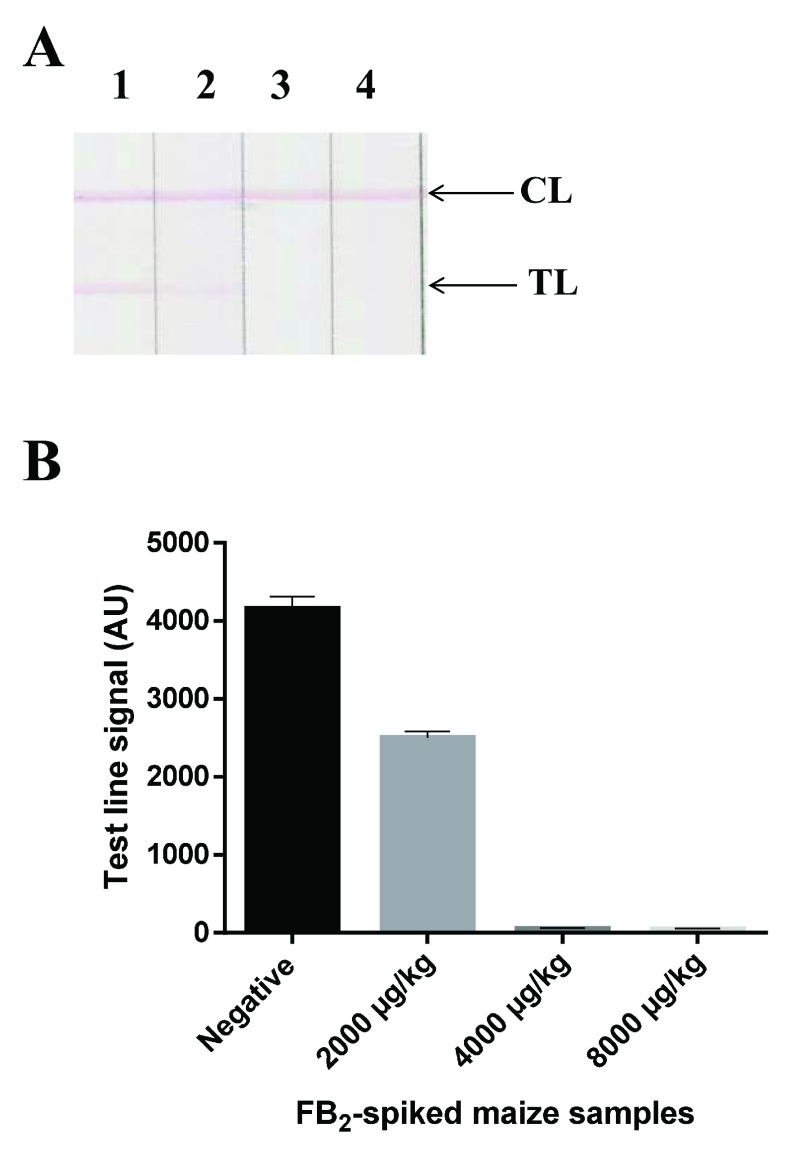
Limit of detection of fumonisin B _2_ (FB _2_) in maize by the developed lateral flow immunoassay. ( **A**) Images of negative controls (1) and diluted extracts of blank maize sample spiked with FB _2_ at 2000 µg/kg (2), 4000 µg/kg (3), and 8000 µg/kg (4) on representative lateral flow immunoassay strips. CL, control line; TL, test line. Experiment was performed with 8 replicates. ( **B**) Quantification of test line signals. AU, arbitrary unit.

Previously, we have shown that the polyclonal IgY antibody used in the present study, recognized FB _1_ and FB _2_ with different affinities (IC _50_ = 10 and 49 ng/ml for FB _1_ and FB _2_ respectively) ( Do *et al.*, 2016). To determine if the developed LFIA could detect FB _2_ in maize at the cut-off level of 4000 µg/kg, this toxin was spiked into a blank sample at 2000 µg/kg, 4000 µg/kg, and 8000 µg/kg. Results ( Figure 6) showed that no visible line was formed at test zone for samples spiked with 4000 µg/kg and 8000 µg/kg of FB _2_. On the contrary, faint signals were still observed at the test line for samples spiked with 2000 µg/kg of FB _2_. Therefore, the limit of detection of our LFIA for FB _2_ was also 4000 µg/kg, meaning that the developed test could be used for screening of total FBs in maize. The extended incubation time between detection conjugate and the toxins (30 minutes) and the optimal amount of labeled antibody are likely able to compensate for the difference in affinity of IgY antibody for FB _1_ and FB _2_.

### Cross-reactivity tests

**Figure 7.**
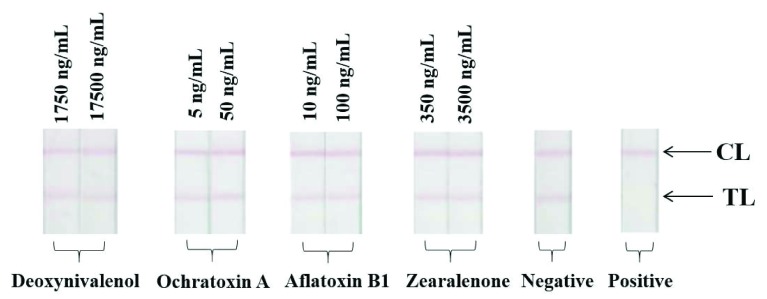
Cross-reactivity of the developed lateral flow immunoassay against deoxynivalenol, ochratoxin A, aflatoxin B1, and zearalenone. Deoxynivalenol (1750 and 17500 ng/mL), ochratoxin A (5 and 50 ng/mL), aflatoxin B1 (10 and 100 ng/mL), zearalenone (350 and 3500 ng/mL) were spiked into diluted extracts of blank maize samples and incubated with detection conjugate for 30 minutes before being analyzed on lateral flow immunoassay (LFIA) test strips. Negative, negative controls; Positive, 40 ng/mL fumonisin B _1_ (FB _1_), CL, control line; TL, test line.

Cross-reactivity tests were performed using deoxynivalenol, ochratoxin A, aflatoxin B1 and zearalenone spiked into blank maize extracts at two different concentration levels (100-fold and 1000-fold of MRL). Figure 7 showed that there was no difference in signal intensities at test line position between negative control and samples spiked with low and high concentrations of the tested mycotoxins. Therefore, the developed LFIA did not cross-react with deoxynivalenol, ochratoxin A, aflatoxin B1 and zearalenone.

### Analysis of naturally contaminated maize samples by IgY LFIA

A total of 19 maize samples were analyzed by HPLC-MS/MS and the developed assay. By HPLC-MS/MS, all samples were negative with FB _2_, while 11 samples were positive with FB _1_, with concentrations ranging from 27 µg/kg to 7850 µg/kg ( Table 1). One sample (No 16: 7850 µg/kg of FB _1_) exceeded the maximum legislative limits (EU) of 4000 µg/kg ( EC, 2007). Notably, only this sample was positive by IgY LFIA. Although the sample size was relatively low, these findings indicated that results by the IgY LFIA were in good agreement with those from MSthe standard method as no false positive or false negative results were found.

**Table 1.** Analysis of naturally contaminated maize samples by high performance liquid chromatography–mass spectrometry (HPLC-MS/MS) method and the IgY Lateral Flow Immunoassay.

Sample	FB _1_ concentration by HPLC-MS/MS (µg/kg)	FB _2_ concentration by HPLC-MS/MS (µg/kg)	Analysis by the developed LFIA
1	1760	Not detected	Negative
2	Not detected	Not detected	Negative
3	183	Not detected	Negative
4	95	Not detected	Negative
5	Not detected	Not detected	Negative
6	Not detected	Not detected	Negative
7	Not detected	Not detected	Negative
8	27	Not detected	Negative
9	129	Not detected	Negative
10	1150	Not detected	Negative
11	Not detected	Not detected	Negative
12	840	Not detected	Negative
13	Not detected	Not detected	Negative
14	Not detected	Not detected	Negative
15	148	Not detected	Negative
16	7850	Not detected	Positive
17	Not detected	Not detected	Negative
18	35	Not detected	Negative
19	322	Not detected	Negative

## Discussion

Fumonisin contamination in maize is now a widely recognized problem and it has been shown to be relevant in many regions of the World, including Vietnam ( Hu *et al.*, 2019; Scussel *et al.*, 2014; Mngqawa *et al.*, 2016; Hieu Phuong *et al.*, 2015). Among fumonisins, the most prevalent that contaminates maize is FB _1_, followed by FB _2_ ( Anfossi *et al.*, 2010; Marasas, 2001). In term of toxicity, fumonisins have been associated with adverse health conditions such as pulmonary edema ( Harrison *et al.*, 1990); liver and nephron damage ( Gelderblom *et al.*, 1988); or liver and esophagus cancers ( Marasas *et al.*, 1988). As a result, international regulations have been adopted to restrict the maximum residue limit of total fumonisins (as the sum of FB _1_ and FB _2_) in raw maize at 4000 µg/kg ( EC, 2007). Conventional analytical methods for fumonisins consist of liquid chromatography coupled with detectors such as UV–Vis spectrophotometry, fluorescence, and mass spectrometry ( Kamle *et al.*, 2019). Although these methods allow quantification of individual fumonisin in food samples, they require complex equipment and need several hours to complete. Consequently, rapid methods for fumonisin analysis such as LFIAs have become increasingly important as they are less expensive, easier to use, very rapid and suitable for on-site analysis. However, LFIAs can only provide qualitative or semi-quantitative results and are therefore recommended for screening purpose ( Zheng *et al.*, 2006). For any positive samples detected by LFIAs, the exact mycotoxin concentration needs to be determined by a confirmatory reference method such as HPLC ( Zheng *et al.*, 2006). 

Several lateral flow immunoassays for fumonisin detection in maize have been reported, with limits of detection (LODs) ranging from 12-1200 µg/kg ( Anfossi *et al.*, 2010; Molinelli *et al.*, 2009; Urusov *et al.*, 2017; Wang *et al.*, 2013). Nevertheless, the low LODs of these assays are not relevant in regard to the adopted MRL of 4000 µg/kg, as they would generate an increased number of false-positive results (according to the adopted MRL) that will be revealed by costly confirmatory methods. This will increase the cost of the whole analytical procedure. To mitigate this problem, some commercial LFIAs require a reader, which allows semi-quantitative analysis of fumonisins in the samples (Reveal ^®^ Q+ from Neogen, Fumo-V from Vicam, AgraStrip® WATEX® from Romer Labs). However, the applicability of these assays is limited in low-resourced environments because of the high cost of the reader. In the present study, we have developed a novel lateral flow immunoassay for total fumonisin (as the sum of FB _1_ and FB _2_) with LOD equal to the MRL of 4000 µg/kg. In the developed assay, a positive sample with a total fumonisin concentration greater than or equal to 4000 µg/kg will result in no visible signal at the test line, while a negative sample with a fumonisin concentration less than or equal to 2000 µg/kg will generate a visible signal at the test line. This would reduce the false positive rate comparing to other LFIAs reported in the literature, while maintaining the simplicity of the assay as the results could be read with the naked eye.

Another novelty of this study is the use of polyclonal IgY antibody for the preparation of detection conjugate in LFIA. To the best of our knowledge, this is the first immunochromatographic test for fumonisins that employs IgY as the principal component. Most LFIAs for fumonisins reported to date are based on monoclonal antibodies that are costly to produce and involves ethical issues of animal welfare ( Dias da Silva & Tambourgi, 2010). We have previously shown that the IC50 values of the IgY used in this study were 10 and 49 ng/mL for FB1 and FB2 respectively ( Do *et al.*, 2016), meaning that the affinities of our IgY towards these mycotoxins are comparable to or even higher than monoclonal antibodies reported in the literature ( Ling *et al.*, 2014; Maragos *et al.*, 2018; Yu & Chu, 1999; Zhang *et al.*, 2018a). Therefore, even though there is no difference in reactant consumption between IgY-based LFIA and IgG-based tests for fumonisin detection, IgY-based LFIA is significantly cheaper due to the abundance of IgY in the egg yolk. We obtained approximately 8 mg of purified IgY from a single egg, which is enough to produce 320000 tests. Of note, IgY in this study was produced in 2016, stored at -80 °C, and used for the development of the assay during the period of 2018–2019, meaning that polyclonal IgY antibody was quite stable over extended periods of time under preservation at -80 °C.

One limitation of the LFIA developed in this study is that the analysis time is longer than those of commercial assays as it requires an incubation step of 30 minutes to allow FB _1_ and FB _2_ to saturate the binding sites of detection antibodies. However, as mentioned above, this additional step was likely able to compensate for the difference in affinity of IgY antibody for FB _1_ and FB _2_, enabling the new assay to meet the cut-off level of 4000 µg/kg for both these toxins.

## Conclusions

In conclusion, this is the first study to use polyclonal IgY antibody in LFIA for simple detection of total fumonisins (as the sum of FB1 and FB2) in maize with the limit of detection equal to the MRL of 4000 µg/kg. The main advantage of this assay is its cost-effectiveness and ability to accurately detect total fumonisins at MRL in maize.

## Data availability

### Underlying data

Figshare: Raw data for "Development of an IgY-based lateral flow immunoassay for detection of fumonisin B in maize". https://doi.org/10.6084/m9.figshare.8320775 ( Tran *et al.*, 2019)

This project contains the following underlying data:

- Dataset 1_Raw scans of lateral flow test strips.rar (Folder includes raw images of lateral flow strips)
- Dataset 2.pptx (PowerPoint file includes HPLC-MS chromatograms of naturally contaminated samples for quantification of FB1 and FB2.)
- Dataset 3.xlsx (Excel file includes quantification of signal intensities of lateral flow test strips and quantification of FB1 and FB2 from HPLC-MS output.)

Data are available under the terms of the Creative Commons Zero "No rights reserved" data waiver (CC0 1.0 Public domain dedication).
