# Supplementary material for: Development of an IgY-based lateral flow immunoassay for detection of fumonisin B in maize
Source: F1000Res. 2019 Dec 12;8:1042. Originally published 2019 Jul 10. [Version 2] doi: 10.12688/f1000research.19643.2 (PMC6950345; doi:10.12688/f1000research.19643.2)
Supplement: Peer review history

# Peer review: Reviewer response for version 2

**DOI:** 10.5256/f1000research.23755.r57741

**Published:** 2020-01-08

**Authors:** Zhanhui Wang

**Affiliations:** 1 Beijing Advanced Innovation Center for Food Nutrition and Human Health, Beijing Key Laboratory of Detection Technology for Animal-Derived Food Safety, China Agricultural University, Beijing, China

I generally agree the author's response to my review and suggest the paper should be accepted after minor revision. I suggest the authors should cited recent and typical references to support their study, for example, from my group. We have tested the cross-reactivity of antibody for FB3 and we have extensively study immunoassay for FB1 detection.

1. Chenglong Li, Tiejun Mi, Gea Oliveri Conti, Qing Yu, Kai Wen, Jianzhong Shen, Margherita Ferrante, Zhanhui Wang*. Development of a screening fluorescence polarization immunoassay for the simultaneous detection of fumonisins B₁ and B₂ in maize. *Journal of Agricultural and Food Chemistry*, 2015, 63, 4940–4946 ^1^.

1. Qing Yu, Heng Li, Chenglong Li, Suxia Zhang, Jianzhong Shen, Zhanhui Wang*. Gold nanoparticles-based lateral flow immunoassay with silver staining for simultaneous detection of fumonisin B1 and deoxynivalenol in maize. *Food Control*, 2015, 54, 347–352 ^2^.
2. **Zhanhui Wang** **, **Heng Li, Chenglong Li, Qing Yu, Jianzhong Shen*, Sarah De Saeger.  Development and application of a quantitative fluorescence-based immunochromatographic assay for fumonisin B _1_ in maize. Journal of Agricultural Food and Chemistry, 2014, 62, 6294–6298 ^3^.
3.  Yajie Sheng, Wenxiao Jiang, Sarah De Saeger, Jianzhong Shen, Suxia Zhang, Zhanhui Wang*. Development of a sensitive enzyme-linked immunosorbent assay for the detection of fumonisin B _1_ in maize. Toxicon, 2012, 60, 1245–1250 ^4^.
4. Chenglong Li, Kai Wen, Tiejun Mi, Xiya Zhang, Huiyan Zhang, Suxia Zhang, Jianzhong Shen, Zhanhui Wang*. A universal multi-wavelength fluorescence polarization immunoassay for multiplexed detection of mycotoxins in maize. *Biosensors & Bioelectronics*, 2016, 79, 258–265 ^5^.

I confirm that I have read this submission and believe that I have an appropriate level of expertise to confirm that it is of an acceptable scientific standard.

## References

[1] : Development of a screening fluorescence polarization immunoassay for the simultaneous detection of fumonisins B₁ and B₂ in maize. *J Agric Food Chem*.2015;63(20) : 10.1021/acs.jafc.5b01845 4940-6 10.1021/acs.jafc.5b01845 25942573

[2] : Gold nanoparticles-based lateral flow immunoassay with silver staining for simultaneous detection of fumonisin B1 and deoxynivalenol. *Food Control*.2015;54: 10.1016/j.foodcont.2015.02.019 347-352 10.1016/j.foodcont.2015.02.019

[3] : Development and application of a quantitative fluorescence-based immunochromatographic assay for fumonisin b1 in maize. *J Agric Food Chem*.2014;62(27) : 10.1021/jf5017219 6294-8 10.1021/jf5017219 24930671

[4] : Development of a sensitive enzyme-linked immunosorbent assay for the detection of fumonisin B₁ in maize. *Toxicon*.2012;60(7) : 10.1016/j.toxicon.2012.08.011 1245-50 10.1016/j.toxicon.2012.08.011 22960014

[5] : A universal multi-wavelength fluorescence polarization immunoassay for multiplexed detection of mycotoxins in maize. *Biosens Bioelectron*.2016;79: 10.1016/j.bios.2015.12.033 258-65 10.1016/j.bios.2015.12.033 26720917

# Peer review: Reviewer response for version 1

**DOI:** 10.5256/f1000research.21541.r52435

**Published:** 2019-09-02

**Authors:** Boris B. Dzantiev

**Affiliations:** 1 A.N. Bach Institute of Biochemistry, Research Centre of Biotechnology, Russian Academy of Sciences (RAS), Moscow, Russian Federation

The first IgY-based lateral flow immunoassay of fumonisin B is presented. The earlier known techniques were based on the IgG use. At whole, the study is well-described and contains all necessary stages of the assay optimization, characterization and validation. However, the necessity of new test is not clear from the manuscript. The common advantages of IgY are widely known, but were not demonstrated for the proposed test system. To clarify this question, the data about reached advantages (lower reactants consumption, higher stability, etc.) of the proposed test in comparison with the known IgG-based test strips for fumonisin B should be added with their experimental / quantitative confirmation.

I confirm that I have read this submission and believe that I have an appropriate level of expertise to confirm that it is of an acceptable scientific standard, however I have significant reservations, as outlined above.

# Response

**Published:** 2019-12-01

**Authors:** Tung Tran

We thank the reviewer for comments and detailed suggestions. We herein clarify issues shown by the reviewer. The manuscript has been rewritten according to the comments, and a discussion part has been added to further explain the need for a new lateral flow immunoassay (LFIA) for total fumonisins (defined as the sum of FB _1_ and FB _2_) with the limit of detection (LOD) equal to the maximum residue limit (MRL) of this mycotoxin group in raw maize and to underline the advantages of IgY for the production of LFIA.

- The first IgY-based lateral flow immunoassay of fumonisin B is presented. The earlier known techniques were based on the IgG use. At whole, the study is well-described and contains all necessary stages of the assay optimization, characterization and validation. However, the necessity of new test is not clear from the manuscript. The common advantages of IgY are widely known, but were not demonstrated for the proposed test system. To clarify this question, the data about reached advantages (lower reactants consumption, higher stability, etc.) of the proposed test in comparison with the known IgG-based test strips for fumonisin B should be added with their experimental / quantitative confirmation.

- The affinities of polyclonal IgY used in this study (IC _50_ = 10 and 49 ng/mL for FB _1_ and FB _2_) towards FB _1_ and FB _2_ are comparable to monoclonal antibodies reported in the literature (Ling et al., 2014; Maragos et al., 2018; Yu & Chu, 1999; Zhang et al., 2018). Therefore, there is likely no difference in reactant consumption between producing IgY-based and IgG-based LFIAs for fumonisin detection. However, IgY can be produced in large quantity at a much lower cost. We obtained approximately 8 mg of purified IgY from one egg, which is enough to produce 320000 tests.

- IgG has been shown to be more stable than IgY under acid, heat-treatment, and Guanidine-HCl denaturation (Shimizu et al., 1992). However, from our experiences, the IgY polyclonal antibody in this study was produced in 2016, stored at -80 ^o^C, and used for the development of the assay during the period of 2018-2019. This shows that polyclonal IgY antibody was quite stable over extended periods of time under preservation at -80 ^o^C.

- On the stability of the IgY-gold nanoparticle conjugates, our unpublished data showed that the conjugate remained stable beyond 6 months when kept at 4 ^o^C in liquid form.

- In available IgG-based LFIAs for fumonisin detection, detection conjugates are dried on conjugate pad, therefore, it is difficult to compare the stability of the developed test with known IgG-based LFIAs.

References

Ling, S., Pang, J., Yu, J., Wang, R., Liu, L., Ma, Y., et al. (2014). Preparation and identification of monoclonal antibody against fumonisin B(1) and development of detection by Ic-ELISA. *Toxicon, 80*, 64-72. doi:10.1016/j.toxicon.2013.12.008

Maragos, C. M., Sieve, K. K., & Busman, M. (2018). Development of antibodies for N-(1-deoxy-D-fructos-1-yl) fumonisin B1 and cross-reaction with modified fumonisins. *World Mycotoxin Journal, 11*(4), 493-502. doi:10.3920/WMJ2018.2308

Shimizu, M., Nagashima, H., Sano, K., Hashimoto, K., Ozeki, M., Tsuda, K., et al. (1992). Molecular Stability of Chicken and Rabbit Immunoglobulin G. *Bioscience, Biotechnology, and Biochemistry, 56*(2), 270-274. doi:10.1271/bbb.56.270

Yu, F.-Y., & Chu, F. S. (1999). Production and Characterization of Monoclonal Antibodies against Fumonisin B1. *Food and Agricultural Immunology, 11*(4), 297-306. doi:10.1080/09540109999681

Zhang, X., Wang, Z., Fang, Y., Sun, R., Cao, T., Paudyal, N., et al. (2018). Antibody Microarray Immunoassay for Simultaneous Quantification of Multiple Mycotoxins in Corn Samples. *Toxins, 10*(10), 415. doi:10.3390/toxins10100415

# Peer review: Reviewer response for version 1

**DOI:** 10.5256/f1000research.21541.r52437

**Published:** 2019-08-23

**Authors:** Zhanhui Wang

**Affiliations:** 1 Beijing Advanced Innovation Center for Food Nutrition and Human Health, Beijing Key Laboratory of Detection Technology for Animal-Derived Food Safety, China Agricultural University, Beijing, China

The paper described a an IgY-based lateral flow immunoassay for detection of fumonisin B in maize. In general, the paper is more like an experimental report than scientific study in my opinion. The paper provided little new information for readers and the commercial products of LFIA for FBs are already available in the market with high performances. All techniques used in the study are conventional and no any improvement was achieved.  

1. The LFIAs for the rapid detection of FBs are reported by using many probes not only gold. The performance of the LFIA developed by the authors in term of sensitivity, specificity and accuracy are not comparable with those of reports.
2. The novelty of the study relied on the first report of usage of IgY in LFIA for FB in my opinion, however, the production of IgY to FB already reported by the authors. The paper should show the advantages and disadvantages of usage of IgY in LFIA. And there are no comparative data with other antibodies like antibody from mouse or rabbit. Exactly, the production of IgY to FB is also a well established techniques.
3. The main body of the manuscript is the optimization of LFIA conditions, the procedure of optimization is necessary for any analytical methods and not the point of the study. 
4. I do not think the IgY used in the study is the first choice since the affinity and specificity of IgY is inferior in comparison with reported antibodies, which may result in inaccuracy determination.
5. The authors should provide the confirmation data for protein conjugates, gold conjugates if they are firstly reported or just cited the reference if these data already reported. For LFIAs, the gold-antibody is important, why the authors have not optimized the preparation of gold-protein conjugates? The low sensitivity of the LFIAs maybe derived from the coating antigens and suggest the authors should evaluate different coating antigens differentiating with ratio, hapten and conjugation methods. In addition, the authors should test the cross-reactivity with FB3 since the analog is available and a potential interferent. 

In conclusion, the paper did not describe a new experimental, observational, or computational method, test or procedure. I have to be against indexing and hope the suggestions could help the authors improve the study.

I confirm that I have read this submission and believe that I have an appropriate level of expertise to state that I do not consider it to be of an acceptable scientific standard, for reasons outlined above.

# Response

**Published:** 2019-12-01

**Authors:** Tung Tran

**General comments:**

We thank the reviewer for comments and detailed suggestions. Please find below our answers for the points raised by the reviewer. The manuscript has been rewritten according to the comments, and a discussion part has been added to further explain the need for a new lateral flow immunoassay (LFIA) for total fumonisins (defined as the sum of FB _1_ and FB _2_) with the limit of detection (LOD) equal to the maximum residue limit (MRL) of this mycotoxin group in raw maize and to underline the advantages of IgY for the production of LFIA.

- The paper described an IgY-based lateral flow immunoassay for detection of fumonisin B in maize. In general, the paper is more like an experimental report than scientific study in my opinion. The paper provided little new information for readers and the commercial products of LFIA for FBs are already available in the market with high performances. All techniques used in the study are conventional and not any improvement was achieved. 

- We agree that commercial Lateral flow immunoassays (LFIAs) for fumonisins B are available. However, most of which are based on monoclonal antibodies that are costly to produce. Several commercial LFIAs for fumonisins require a reader to obtain accurate results, which limits their applicability in low-resourced environments.

- In this study, we developed a new polyclonal IgY-based LFIA, which significantly reduces the cost of production. Furthermore, result interpretation is based on complete test line disappearance, instead of a decreased intensity of test line color. Thus, results could be read with the naked eye.

- Another novelty of this study is that the developed LFIA was optimized to have the limit of detection for total fumonisins equal to the maximum residue limit adopted by the Commission of the European Communities, which would reduce the false positive rate comparing to other LFIAs reported in the literature, while maintaining the simplicity of the assay as the results could be read with the naked eye

 

- The LFIAs for the rapid detection of FBs are reported by using many probes not only gold. The performance of the LFIA developed by the authors in term of sensitivity, specificity, and accuracy are not comparable with those of reports.

- We agree that other research groups have successfully applied probes other than gold nanoparticles, which increased the sensitivity of their LFIAs. However, in this case, the developed test is intended for screening of raw maize with LOD equal to the MRL (4000 µg/kg for the sum of FB _1_ and FB _2_) adopted by the Commission of the European communities. Of note, this maximum level is particularly high compared to MRLs of other mycotoxins in cereals (5 µg/kg for ochratoxin A, 4 µg/kg for total aflatoxins…) (EC, 2006). For that reason, we chose gold nanoparticles, which have high stability, as probes (Guerrini et al., 2018; Sajid et al., 2015).

- In terms of accuracy, we have performed analyses of naturally contaminated maize samples by both the developed IgY LFIA and HPLC-MS/MS method and found perfect agreement between the two assays. One positive sample by LFIA was confirmed, by HPLC-MS/MS, to be contaminated with FB _1_ at the level higher than 4000 µg/kg.

 

- The novelty of the study relied on the first report of usage of IgY in LFIA for FB in my opinion, however, the production of IgY to FB already reported by the authors. The paper should show the advantages and disadvantages of usage of IgY in LFIA. And there are no comparative data with other antibodies like antibody from mouse or rabbit. Exactly, the production of IgY to FB is also a well established techniques.

- Indeed, this is the first report of application of IgY in development of LFIA for total fumonisins. In our previous study, IC _50_ values of the obtained IgY were 10 and 49 ng/mL for FB _1_ and FB _2 _respectively (Do et al., 2016). These results show that the affinities of our IgY towards these mycotoxins are comparable to or even higher than some monoclonal antibodies reported in the literature (Ling et al., 2014; Maragos et al., 2018; Yu & Chu, 1999; Zhang et al., 2018).

- As mentioned in the manuscript, the main advantage of using IgY in LFIAs for total fumonisins is cost-effectiveness. From one egg of the immunized hen, we obtained approximately 8 mg of purified IgY, which is enough to produce 320000 tests.

 

- The main body of the manuscript is the optimization of LFIA conditions, the procedure of optimization is necessary for any analytical methods and not the point of the study. 

- The aim of the present study is to develop a new LFIA for total fumonisins with LOD equal to MRL of these toxins in raw maize, whose performance, LOD and cross-reactivity pattern rely on selection and optimization of test components. Therefore, it is necessary to perform and report optimization experiments.

 - Other researchers also demonstrated optimization results in their reports of development of LFIAs for detection of fumonisins (Anfossi et al., 2010; Hao et al., 2018; Wang et al., 2013)

 

- I do not think the IgY used in the study is the first choice since the affinity and specificity of IgY is inferior in comparison with reported antibodies, which may result in inaccuracy determination.

- Once again, with the MRL at 4000 µg/kg, we do not need a test with very low LOD.

- In term of test’s accuracy, mycotoxins are low-molecular weight molecules, thus, they need to be conjugated with carrier proteins to act as immunogens. Therefore, cross-reactivity with other mycotoxins is not possible, even though certain levels of mycotoxins may be present in the chicken feeds.

- Our unpublished data also showed an absence of cross-reactivity of IgY against BSA as KLH-FB _1_ was used for antibody production while BSA-FB _1_ was immobilized on LFIA membranes. 

 

- The authors should provide the confirmation data for protein conjugates, gold conjugates if they are firstly reported or just cited the reference if these data already reported. For LFIAs, the gold-antibody is important, why the authors have not optimized the preparation of gold-protein conjugates? The low sensitivity of the LFIAs maybe derived from the coating antigens and suggest the authors should evaluate different coating antigens differentiating with ratio, hapten and conjugation methods. In addition, the authors should test the cross-reactivity with FB3 since the analog is available and a potential interferent.

- We agree with the reviewer that optimization of gold nanoparticle-antibody is a crucial step for increasing the sensitivity of LFIA, which is relevant in developing an assay that detect contaminants with low MRL, such as Aflatoxin. However, in this study, we aimed to develop a LFIA that detect fumonisins with LOD of 4000 µg/kg, therefore, gold nanoparticle-antibody conjugation was performed as recommended by the manufacturer to ensure the stability of the conjugate.

- We agree that cross-reactivity with FB _3_ should be tested. However, we currently do not have access to standards of FB _3_. Additionally, all LFIAs reported in the literature did not evaluate the cross-reactivity with FB _3_. Furthermore, according to the Commission of the European communities, the maximum residue limit of fumonisins in raw maize accounts for the sum of FB _1_ and FB _2_ only.

Anfossi, L., Calderara, M., Baggiani, C., Giovannoli, C., Arletti, E., & Giraudi, G. (2010). Development and application of a quantitative lateral flow immunoassay for fumonisins in maize. *Anal Chim Acta, 682*(1-2), 104-109. doi:10.1016/j.aca.2010.09.045

Guerrini, L., Alvarez-Puebla, R. A., & Pazos-Perez, N. (2018). Surface Modifications of Nanoparticles for Stability in Biological Fluids. *Materials (Basel, Switzerland), 11*(7), 1154. doi:10.3390/ma11071154

Hao, K., Suryoprabowo, S., Hong, T., Song, S., Liu, L., Zheng, Q., et al. (2018). Immunochromatographic strip for ultrasensitive detection of fumonisin B1. *Food and Agricultural Immunology, 29*(1), 699-710. doi:10.1080/09540105.2018.1439455

Ling, S., Pang, J., Yu, J., Wang, R., Liu, L., Ma, Y., et al. (2014). Preparation and identification of monoclonal antibody against fumonisin B(1) and development of detection by Ic-ELISA. *Toxicon, 80*, 64-72. doi:10.1016/j.toxicon.2013.12.008

Maragos, C. M., Sieve, K. K., & Busman, M. (2018). Development of antibodies for N-(1-deoxy-D-fructos-1-yl) fumonisin B1 and cross-reaction with modified fumonisins. *World Mycotoxin Journal, 11*(4), 493-502. doi:10.3920/WMJ2018.2308

Sajid, M., Kawde, A.-N., & Daud, M. (2015). Designs, formats and applications of lateral flow assay: A literature review. *Journal of Saudi Chemical Society, 19*(6), 689-705. doi:https://doi.org/10.1016/j.jscs.2014.09.001

Wang, Y., Shi, Y.-B., Zou, Q., Sun, J.-H., Chen, Z.-F., Wang, H.-a., et al. (2013). Development of a rapid and simultaneous immunochromatographic assay for the determination of zearalenone and fumonisin B1 in corn, wheat and feedstuff samples. *Food Control, 31*, 180–188. doi:10.1016/j.foodcont.2012.09.048

Yu, F.-Y., & Chu, F. S. (1999). Production and Characterization of Monoclonal Antibodies against Fumonisin B1. *Food and Agricultural Immunology, 11*(4), 297-306. doi:10.1080/09540109999681

Zhang, X., Wang, Z., Fang, Y., Sun, R., Cao, T., Paudyal, N., et al. (2018). Antibody Microarray Immunoassay for Simultaneous Quantification of Multiple Mycotoxins in Corn Samples. *Toxins, 10*(10), 415. doi:10.3390/toxins10100415

# Peer review: Reviewer response for version 1

**DOI:** 10.5256/f1000research.21541.r51089

**Published:** 2019-08-14

**Authors:** Venkataramana Mudili

**Affiliations:** 1 Lorven Biologics Pvt. Ltd., Hyderabad, India

The present manuscript submitted by *Tien et al.* is a very interesting study in current food contaminants aspect. However, my main criticism goes to the detection limits of assay and the target selection in the study.

 As Fumonisins are a group of toxins, out of  which fumonisin was target in the present study was not clear, for example among the fumonisin FB1 is more potent and frequently reported one in may kind of food grains mostly in wheat and maize and the cross reactivity patterns with group of Fusarium toxins need to be studied for example DON, T-2 toxin, Nivalenol etc.

- Introduction part is too vague, need to be improved with recent and relevant references.
- Preparation of IgY-conjugated gold nano particles is not clear, it's totally confusing, authors should clear draft the protocol in a supplementary section or main text.
- Ethical committee guidelines and permission to carry out the study need be given the manuscript.
- ***Selection of nitrocellulose membrane: its very crucial in developing the LFA assay, in the present study sufficient information was not presented by the authors in this section. please be give the clear protocol how its been chosen.***
- OD 4000ug/kg is very high, many studies reported that the LD is under nano gram levels, this need to be further improved for their sensitivity.
- Over study looks good and can be indexed in this journal after substantial revision of the manuscript. 

I confirm that I have read this submission and believe that I have an appropriate level of expertise to confirm that it is of an acceptable scientific standard, however I have significant reservations, as outlined above.

# Response

**Published:** 2019-12-01

**Authors:** Tung Tran

**General comments:**

We thank the reviewer for comments and detailed suggestions. We herein clarify issues shown by the reviewer. The manuscript has been rewritten according to the comments, and a discussion part has been added to further explain the need for a new lateral flow immunoassay (LFIA) for total fumonisins (defined as the sum of FB _1_ and FB _2_) with the limit of detection (LOD) equal to the maximum residue limit (MRL) of this mycotoxin group in raw maize and to underline the advantages of IgY for the production of LFIA.

- As Fumonisins are a group of toxins, which fumonisin was target in the present study was not clear, for example, among the fumonisin, FB1 is more potent and frequently reported one in many kinds of food grains, mostly in wheat and maize.

- In the present study, the targets are Fumonisin B _1_ and Fumonisin B _2_ (FB _1_ and FB _2_) as they are the most frequently reported fumonisins in foods in general, and in raw maize in particular (Abbas, 2006; D’Arco et al., 2009; Magro et al., 2011; Yazar & Omurtag, 2008)

- Besides, according to the Regulation No 1126/2007 of the Commission of the European communities, the maximum residue limit of fumonisins in raw maize (defined as sum of FB1 and FB2) is fixed at 4000 µg/kg (EC, 2007).

 

- The cross reactivity patterns with group of Fusarium toxins need to be studied for example DON, T-2 toxin, Nivalenol etc.

- In the manuscript, we have showed an absence of cross-reactivity of the developed test with deoxynivalenol, ochratoxin A, aflatoxin B1 and zearalenone.

- DON, T-2 toxin, Nivalenol, which were suggested by the reviewer, belong to the same group of trichothecenes, and have similar structure (McCormick et al., 2011). Therefore, we performed only the cross-reactivity test using DON, which has been shown to be the most prevalent trichothecenes in cereals (Foroud et al., 2019; Payros et al., 2016).

 

- Introduction part is too vague, need to be improved with recent and relevant references.

- We thank the reviewer for raising the point, the introduction has been modified and updated with recent literatures.

 

- Preparation of IgY-conjugated gold nano particles is not clear, it's totally confusing, authors should clear draft the protocol in a supplementary section or main text.

- The principle of conjugate preparation has been added to the manuscript and the protocol has been rewritten for better clarity.

 

- Ethical committee guidelines and permission to carry out the study need to be given the manuscript.

- We have provided, in this revision of the manuscript, an ethics approval for animal use for this study.

 

- Selection of nitrocellulose membrane: it’s very crucial in developing the LFA assay, in the present study, sufficient information was not presented by the authors in this section. please be give the clear protocol how it’s been chosen.

- We thank the reviewer for raising the point. Details on flow rates of the membranes have been added as follows:

“ *CNPC-SS12, 10 µm with wicking time of 140 ± 28s/40 mm (MDI technologies, Cat Nº CNPC-SS12-10µm-25mm), UniSart® CN140 with wicking time of 95-155s/ 40 mm (Sartorius, Cat Nº 1UN14ER100025NTB), and UniSart® CN 95 with wicking time of 65-115s/ 40 mm (Sartorius, Cat Nº 1UN95ER100040WS)*”.

- Furthermore, according to the manufacturers, CNPC-SS12-10µm is recommended for environmental and agriculture analytes; CN95 is recommended when a quick response is desirable; whereas CN140 is recommended when high sensitivity is required.

 

- LOD 4000ug/kg is very high, many studies reported that the LOD is under nano gram levels, this need to be further improved for their sensitivity.

- We thank the reviewer for raising this point. It is certainly feasible to optimize the assay to achieve a lower limit of detection. However, the LOD was set at 4000 µg/kg as it is the maximum residue limit (MRL) adopted by the Commission of the European communities. Similar to other lateral flow immunoassays for detection of mycotoxins in foods, this assay is intended for quick screening, and positive results need to be confirmed by instrumental analytical techniques, such as HPLC, LC/MS. Consequently, if the sensitivity of the assay is in the range of nanograms, we will obtain a lot of false-positive results (according to the adopted MRL) that will be revealed by costly confirmatory methods. This will increase the cost of the whole analytical procedure.

References

Abbas, H. K. (2006). Aflatoxin and fumonisin contamination of corn (maize, Zea mays) hybrids in Arkansas. *Crop protection, v. 25*(no. 1), pp. 1-9-2006 v.2025 no.2001.

D’Arco, G., Fernández-Franzón, M., Font, G., Damiani, P., & Mañes, J. (2009). Survey of fumonisins B1, B2 and B3 in conventional and organic retail corn products in Spain and Italy and estimated dietary exposure. *Food Additives & Contaminants: Part B, 2*(2), 146-153. doi:10.1080/02652030903148314

EC, European Commission (2007). Commission Regulation (EC) no 1126/2007 of 28 September 2007 setting maximum levels for certain contaminants in foodstuffs as regards Fusarium toxins in maize and maize products. Official Journal of the European Union, L, 255,14–17.

Foroud, N. A., Baines, D., Gagkaeva, T. Y., Thakor, N., Badea, A., Steiner, B., et al. (2019). Trichothecenes in Cereal Grains – An Update. *11*(11), 634.

Magro, S. L., Campaniello, M., Nardiello, D., & Muscarella, M. (2011). Assessment of Fumonisins B1 and B2 Levels in Commercial Maize-Based Food Products by Liquid Chromatography with Fluorimetric Detection and Postcolumn Chemical Derivatization. *J Food Sci, 76*(1), T1-T4. doi:10.1111/j.1750-3841.2010.01948.x

McCormick, S. P., Stanley, A. M., Stover, N. A., & Alexander, N. J. (2011). Trichothecenes: from simple to complex mycotoxins. *Toxins, 3*(7), 802-814. doi:10.3390/toxins3070802

Payros, D., Alassane-Kpembi, I., Pierron, A., Loiseau, N., Pinton, P., & Oswald, I. P. (2016). Toxicology of deoxynivalenol and its acetylated and modified forms. *Arch Toxicol, 90*(12), 2931-2957. doi:10.1007/s00204-016-1826-4

Yazar, S., & Omurtag, G. Z. (2008). Fumonisins, trichothecenes and zearalenone in cereals. *International journal of molecular sciences, 9*(11), 2062-2090. doi:10.3390/ijms9112062
